# CEACAM1 promotes vascular aging processes

**DOI:** 10.18632/aging.102868

**Published:** 2020-02-21

**Authors:** Florian Kleefeldt, Uwe Rueckschloss, Süleyman Ergün

**Affiliations:** 1 Institute of Anatomy and Cell Biology, Julius-Maximilians-University Würzburg, Würzburg, Germany

**Keywords:** endothelial signaling and barrier, vascular inflammation, aging, CEACAM1, TNFα

Cardiovascular diseases like myocardial infarction and stroke are the leading cause of mortality and morbidity worldwide. Increasing age is an independent risk factor for these diseases. Therefore, therapeutic manipulation of mechanisms that lead to age-dependent pathological vascular alterations like increased intimal thickening, vascular stiffness and enhanced vascular inflammation would be a promising way in order to prevent cardiovascular events. Many pathways involved in these age-related processes are characterized. However, most of them are not suitable targets for pharmaceutical manipulation since their inhibition causes significant adverse effects. Therefore, novel targets are required to modulate age-dependent processes.

Carcinoembryonic antigen (CEA)-related cell adhesion molecule 1 (CEACAM1, CD66a) is a glycoprotein of the immunoglobulin superfamily [1]. Concerning the vasculature, CEACAM1 contributes to angiogenesis by induction of vascular sprouting, promotes morphogenesis and is involved in initial endothelial barrier establishment [2,3]. Interestingly, CEACAM1 is not detectable in quiescent endothelium whereas it is upregulated in angiogenicly activated endothelial cells [2,3]. This again indicates the impact of CEACAM1 on vascular developmental processes. However, CEACAM1 has not been associated with the vascular aging process until recently.

It has been known for a long time that the pro-inflammatory cytokine TNF-α is upregulated within the wall of aging vasculature and contributes to endothelial dysfunction that in turn predicts cardiovascular events. Since we showed previously that CEACAM1 is critically involved in TNF-α-mediated endothelial barrier breakdown via adherens junction disassembly [3], we wondered whether CEACAM1 might also contribute to vascular aging.

As a first hint, we observed re-expression of CEACAM1 in the murine and human vasculature with progressive age [1]. This upregulation of vascular CEACAM1 expression is of great importance since we demonstrated that the presence of CEACAM1 is necessary for age-associated vascular upregulation of TNF-α using a murine CEACAM1 knockout model. Reversely, TNF-α induced the expression of CEACAM1 in cultured endothelial cells, indicating the establishment of a vicious cycle within aging vessels [1]. This interplay with TNF-α also suggests that CEACAM1 might be additionally involved in extravascular age-associated pathologies like rheumatism-related diseases.

A hallmark of vascular aging is the increased deposition of collagen fibers within the media of larger vessels. Intriguingly, we found that only in the presence of CEACAM1 vascular aging in mice was accompanied by vascular fibrosis presumably due to enhanced TGF-β/TGF-βR1 signaling whereas genetic deletion of CEACAM1 completely prevented aortic collagen accumulation [1]. Our *in vitro* data suggest that CEACAM1 modulates endothelial TGF-β/TGF-βR1 signaling in a TNF-α-dependent manner [1]. This contrasts with a previous report showing liver fibrosis due to genetic ablation of CEACAM1. However, hepatic fibrosis was attributed to hepatic steatosis due to impaired insulin clearance in CEACAM1 knockout mice [4]. It indicates that CEACAM1 influences TGF-β-mediated processes and diseases like fibrosis in an organ-specific manner.

Nevertheless, age-related CEACAM1-dependent vascular collagen accumulation might increase arterial stiffness, which is known to augment cardiac afterload permanently resulting in concentric ventricular hypertrophy and cardiomyopathy. It was reported that metabolic changes due to hyperinsulinemia cause cardiac hypertrophy and reduced cardiac performance in younger CEACAM1 knockout mice. However, the CEACAM1-dependent vascular fibrosis-related hemodynamic effects could affect cardiac morphology more profoundly resulting in aggravated hypertrophy in the wild type at advanced age. Whether CEACAM1 deficiency protects mice from age-related cardiac hypertrophy needs to be elucidated.

Finally, we found that CEACAM1 contributes to the age-related increase in oxidative stress within the vasculature which promotes endothelial barrier impairment by HIF-1α stabilization and subsequent VEGF/VEGFR-2 signaling [3,5,6,7]. It is well-known that oxidative stress is also critically involved in processes that promote angiopathies like atherosclerosis [8]. Although there are some hints pointing to a role of CEACAM1 in atherosclerosis the exact contribution of CEACAM1 in these processes is yet to be defined [3,7].

**Figure 1:**
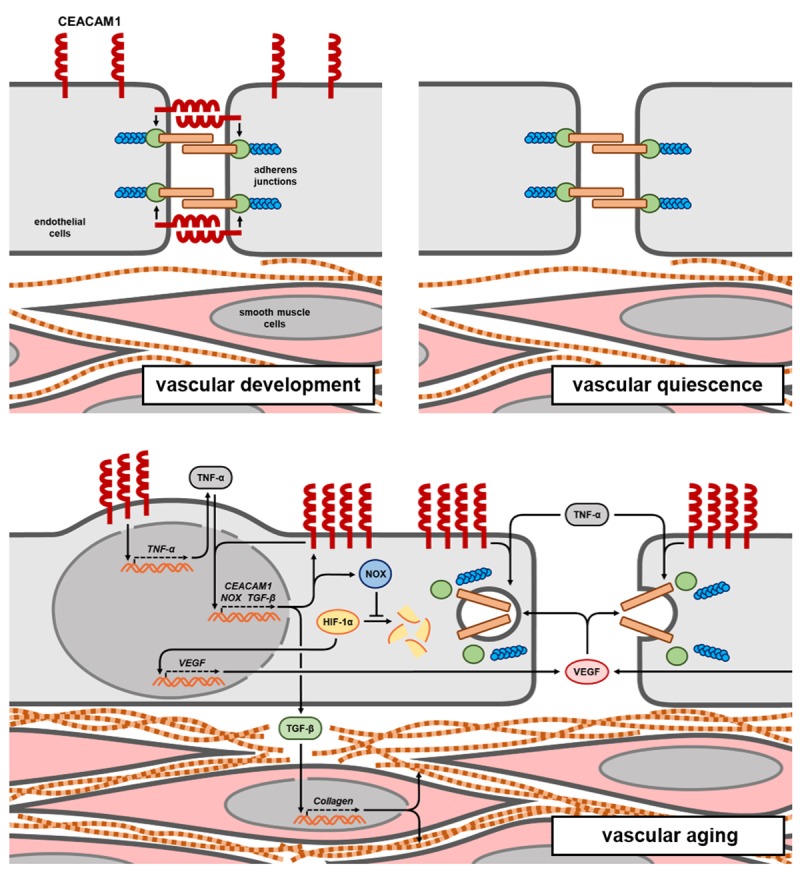
**Vascular function of CEACAM1 during lifetime.** Early in life, moderate endothelial expression of CEACAM1 contributes to vessel maturation, i.e. establishment of the endothelial barrier by promoting adherens junction formation. Thereafter, in quiescent vessels CEACAM1 expression is almost completely downregulated. However, during vascular aging, a remarkable re-expression of CEACAM1 critically contributes to upregulation of proinflammatory TNF-α, to endothelial barrier impairment and vascular collagen deposition.

In summary, CEACAM1 seems to be a Janus-faced protein with respect to its vascular function (Figure 1). Early in life, CEACAM1 expression in developing vessels substantially promotes vascular sprouting, morphogenesis and endothelial barrier implementation. However, with advancing age vascular CEACAM1 expression increases and, presumably due to its interplay with TNF-α, significantly contributes to main characteristics of aging vessels, i.e. increased inflammatory signaling, impaired endothelial barrier function and collagen deposition within the vascular wall. Thus, we identified CEACAM1 as an important player in the process of vascular aging. Identification of mechanisms of vascular aging in detail that are regulated by endothelial and vascular presence of CEACAM1 might therefore open up new therapeutic strategies to slow-down the vascular aging process thereby reducing the risk of life-threatening cardiovascular events.
